# Circulating CTRP-1 levels are elevated in women with polycystic ovary syndrome: A case–control study

**DOI:** 10.1371/journal.pone.0356461

**Published:** 2026-08-25

**Authors:** Cavide Ali, Alper Şayir, Feride Öçba, Deniz Öztürk Atan, Melike Sevde Harmancı, Mustafa Cem Sürecek, Hacer Cavidan Gülerman

**Affiliations:** 1 Department of Obstetrics and Gynecology, Ankara Bilkent City Hospital, Ankara, Türkiye; 2 Obstetrics and Gynecology Clinic, Kadıköy Acıbadem Hospital, Istanbul, Turkey; 3 Department of Obstetrics and Gynecology, Çanakkale Onsekiz Mart University, Çanakkale, Türkiye; Weill Cornell University, UNITED STATES OF AMERICA

## Abstract

**Objective:**

To investigate circulating C1q/TNF-related protein-1 (CTRP-1) levels in women with polycystic ovary syndrome (PCOS) and evaluate its potential association with metabolic and reproductive features of the syndrome.

**Methods:**

A prospective cross-sectional case–control study was conducted between October 2021 and March 2022 including 96 women (48 with PCOS and 48 controls) aged 15–40 years. PCOS was diagnosed according to the 2003 Rotterdam criteria. Anthropometric measurements and fasting venous blood samples were obtained during the early follicular phase. Serum CTRP-1 levels were measured using enzyme-linked immunosorbent assay (ELISA). Metabolic and hormonal parameters, including HOMA-IR, AMH, and androgen levels, were analyzed. Receiver operating characteristic (ROC) analysis and analysis of covariance (ANCOVA) adjusted for age and body mass index (BMI) were performed.

**Results:**

Serum CTRP-1 levels were significantly higher in women with PCOS compared with controls (54.9 ± 5.2 vs. 26.6 ± 4.8 ng/mL, p < 0.001). CTRP-1 concentrations were associated with several metabolic and hormonal parameters, including BMI, fasting insulin, triglyceride levels, HDL cholesterol, and AMH. ROC curve analysis demonstrated excellent discriminatory ability within this cohort, although these findings should be interpreted cautiously given the relatively small sample size and case–control design. In multivariable analysis adjusting for age and BMI, CTRP-1 remained independently associated with PCOS (β = 28.97, p < 0.001).

**Conclusion:**

Circulating CTRP-1 levels are elevated in women with PCOS and appear to be associated with both metabolic and reproductive characteristics of the syndrome. CTRP-1 may represent a potential biomarker related to metabolic dysregulation in PCOS; however, larger prospective and multicenter studies are required to validate its clinical and diagnostic utility.

## 1 Introduction

Polycystic ovary syndrome (PCOS) is a common endocrine and metabolic disorder characterized by oligo- anovulation or chronic anovulation, hyperandrogenism, and polycystic ovarian morphology on ultrasonography. Affecting approximately 6–10% of women of reproductive age, PCOS is one of the most common endocrine disorders in this population [1,2]. The clinical importance of PCOS extends beyond reproductive dysfunction, as the syndrome is frequently associated with metabolic complications including obesity, type 2 diabetes mellitus, dyslipidemia, hypertension, and cardiovascular disease [3–5]. PCOS is a heterogeneous condition with diverse clinical manifestations resulting from different combinations of endocrine and metabolic abnormalities.

Diagnostic criteria for PCOS have evolved over time. The first formal definition was introduced in 1990 by the National Institute of Child Health and Human Development (NICHD), emphasizing hyperandrogenism and menstrual dysfunction after exclusion of related endocrinopathies [6]. In 2003, the Rotterdam consensus expanded the criteria to include polycystic ovarian morphology, requiring the presence of at least two of the following: oligo-anovulation, clinical or biochemical hyperandrogenism, and polycystic ovarian morphology on ultrasonography [7]. Later, the Androgen Excess Society proposed a modified definition emphasizing androgen excess as a central feature of the syndrome [8,9]. More recently, the 2018 International Evidence-Based Guideline for the Assessment and Management of PCOS provided updated recommendations aimed at improving diagnostic consistency and clinical management [10].

Insulin resistance is considered a key component in the pathophysiology of PCOS and contributes to both metabolic and reproductive abnormalities. In recent years, increasing attention has been directed toward adipokines involved in energy metabolism and insulin signaling pathways. Among these, the C1q/TNF-related protein (CTRP) family has emerged as a group of adipokines structurally related to adiponectin and involved in the regulation of glucose metabolism, lipid homeostasis, inflammation, and insulin sensitivity [11–13]. Alterations in different CTRP family members have been reported in several metabolic conditions, including obesity, type 2 diabetes mellitus, cardiovascular disease, hepatic steatosis, and metabolic syndrome.

The CTRP protein family shares a conserved structural organization consisting of an N-terminal signal peptide, a short variable region, a collagen-like domain, and a C-terminal globular domain homologous to complement component C1q [14]. These proteins primarily form biologically active homotrimers, although heterotrimeric structures have also been described.

Previous studies investigating CTRP family members in PCOS remain limited. Jerobin et al. reported no significant differences in CTRP-2 and CTRP-9 levels between women with PCOS and healthy controls [15], whereas Gülümsek et al. demonstrated significantly lower CTRP-3 levels in patients with PCOS compared with controls [16]. However, to the best of our knowledge, circulating CTRP-1 levels have not yet been specifically investigated in women with PCOS.

CTRP-1 is expressed in multiple tissues, including adipose tissue, placenta, ovaries, liver, skeletal muscle, kidneys, and cardiac muscle [11]. Experimental studies suggest that CTRP-1 regulates glucose uptake through Akt-mediated translocation of GLUT-4 in muscle cells, promotes fatty acid oxidation via MAPK signaling pathways, and modulates lipid metabolism in the liver. Furthermore, CTRP-1 has been implicated in anti-inflammatory processes and metabolic regulation through activation of AMP-activated protein kinase (AMPK) and MAPK signaling pathways [13,17–21]. These pathways are closely linked to insulin resistance and metabolic inflammation, two central mechanisms in the pathophysiology of PCOS. Elevated circulating CTRP-1 levels have been reported in several metabolic disorders, including type 2 diabetes, prediabetes, coronary artery disease, congestive heart failure, and atherosclerosis, suggesting a potential role in metabolic and inflammatory regulation [22]. In the context of PCOS, elevated CTRP-1 levels may represent a compensatory metabolic response to insulin resistance, chronic low-grade inflammation, and adipose tissue dysfunction, although the precise biological mechanisms remain incompletely understood.

Given the central role of insulin resistance and chronic low-grade inflammation in PCOS, adipokines involved in metabolic signaling pathways may provide important insights into its pathophysiology. However, data regarding CTRP-1 in PCOS are currently lacking. Considering its regulatory effects on glucose metabolism, lipid homeostasis, and inflammatory pathways, CTRP-1 represents a biologically plausible mediator linking metabolic dysfunction with reproductive abnormalities in PCOS. Therefore, the present study aimed to investigate circulating CTRP-1 levels in women with PCOS and to explore their potential clinical and metabolic associations in relation to hormonal and metabolic parameters.

## 2 Materials and methods

### 2.1 Study design and objectives

This prospective cross-sectional case–control study aimed primarily to determine circulating CTRP-1 levels in women with polycystic ovary syndrome (PCOS) and healthy controls, evaluate the potential diagnostic utility of CTRP-1 for PCOS, and assess its relationship with insulin resistance. In addition, the study aimed to explore the potential role of CTRP-1 in the pathophysiology of PCOS. Secondary objectives included examining the influence of demographic and anthropometric parameters (age, body weight, and body mass index [BMI]) as well as biochemical and metabolic variables (fasting glucose, insulin, HOMA-IR [Homeostatic Model Assessment of Insulin Resistance], DHEA-S [Dehydroepiandrosterone sulfate], SHBG [Sex Hormone-Binding Globulin], AMH [Anti-Müllerian Hormone], and lipid profile) on CTRP-1 levels.

Ethical approval was obtained from the Academic Committee (08 June 2021, decision no. 20) and the Ethics Committee of Health Sciences University Ankara Bilkent City Hospital (29 September 2021, decision no. E2-21–853). The study was conducted in accordance with the Declaration of Helsinki. Written informed consent was obtained from all participants.Participants were recruited, and data were collected between 01 October 2021 and 31 March 2022.Sample size calculation based on the study by Majidi et al. indicated that 48 participants per group would provide 80% statistical power with an alpha level of 0.05 and a beta of 0.2.

### 2.2 Participants

#### 2.2.1 PCOS group.

Women aged 15–40 years who were diagnosed with PCOS according to the 2003 Rotterdam criteria were included. The diagnosis required the presence of at least two of the following features: oligo- or anovulation, clinical or biochemical hyperandrogenism, and polycystic ovarian morphology on ultrasonography. Menstrual irregularity was defined as a cycle length shorter than 21 days or longer than 35 days. Hirsutism was assessed using the modified Ferriman–Gallwey scoring system, with a score ≥8 considered diagnostic. Biochemical hyperandrogenemia was defined based on total testosterone, free testosterone, and the Free Androgen Index (FAI > 5).

#### 2.2.2 Control group.

The control group consisted of women aged 15–40 years who presented for routine gynecological evaluation and had regular menstrual cycles, normal ovarian morphology on ultrasonography, and no clinical or biochemical evidence of hyperandrogenism. None of the control participants fulfilled the Rotterdam diagnostic criteria for PCOS. Participants with known endocrine, metabolic, or systemic disorders were excluded from the control group. Although some participants were recruited from the same outpatient clinical setting, women with suspected PCOS, menstrual dysfunction, infertility related to ovulatory disorders, or clinical hyperandrogenism were not included as controls.

#### 2.2.3 Exclusion criteria.

Participants were excluded if they were outside the age range of 15–40 years, declined participation, or had systemic chronic diseases (e.g., hypertension, liver disease, or renal dysfunction) or endocrine disorders such as adrenal or thyroid disease.

### 2.3 Study protocol

Detailed medical history, anthropometric measurements, menstrual patterns, fertility characteristics, and medication use were recorded for all participants. Venous blood samples (~10 mL) were collected on days 2–3 of the menstrual cycle after an overnight fast of at least 8 hours. Serum samples were separated by centrifugation at 4000 rpm for 10 minutes and stored at −80°C until analysis. After recruitment was complete, all samples were assayed together in a single analytical run on the same day, in the same laboratory, using the same ELISA kit lot, thereby minimizing inter-assay and batch-related variability.

Serum CTRP-1 concentrations were measured using a commercially available enzyme-linked immunosorbent assay (ELISA) kit (CLOUD-CLONE CORP., USA; catalog no. SEK170Hu), with optical density measured at 450 nm. The limit of detection (LOD) for the assay was 8.3 ng/mL. Other biochemical parameters, including total and free testosterone, SHBG, FSH, LH, estradiol, AMH, fasting glucose and insulin, HbA1c, and lipid profile, were obtained from hospital laboratory records. Insulin resistance was assessed using the HOMA-IR.

### 2.4 Statistical analysis

Statistical analyses were performed using SPSS version 22.0 (IBM Corp., Armonk, NY, USA). The normality of data distribution was assessed using the Kolmogorov–Smirnov test together with visual inspection of histograms and box plots. Continuous variables were presented as mean ± standard deviation or median (minimum–maximum) according to data distribution. Normally distributed variables were compared using Student’s t-test, whereas non-normally distributed variables were analyzed using the Mann–Whitney U test. Comparisons involving more than two groups were performed using one-way analysis of variance (ANOVA) with Bonferroni-corrected post hoc analyses where appropriate. Categorical variables were compared using Pearson’s chi-square test, Fisher’s exact test, or the Fisher–Freeman–Halton test, as applicable.

Receiver operating characteristic (ROC) curve analysis was performed to evaluate the discriminatory ability of CTRP-1 for identifying PCOS within the present cohort. The area under the curve (AUC), sensitivity, specificity, and optimal cut-off values were calculated with 95% confidence intervals (Clopper–Pearson method for sensitivity and specificity). Internal validation was performed using 2000-iteration bootstrap resampling and repeated five-fold cross-validation, and an optimism-corrected AUC was estimated using the Harrell bootstrap procedure. The incremental discriminatory value of CTRP-1 was further assessed by comparing logistic regression models based on age and BMI with and without CTRP-1 using cross-validated AUC. Because the ROC analysis was both derived and evaluated within the same dataset, the resulting diagnostic performance estimates should be interpreted cautiously and considered preliminary.

Correlation analyses were performed to evaluate associations between CTRP-1 levels and demographic, anthropometric, metabolic, and hormonal variables. Because multiple exploratory correlation analyses were conducted, findings with borderline statistical significance were interpreted cautiously. Correlation analyses were additionally corrected for multiple comparisons using the Benjamini–Hochberg false discovery rate (FDR) procedure, and both unadjusted and FDR-adjusted p-values were considered when interpreting the findings.

Because age and BMI differed significantly between the PCOS and control groups, an analysis of covariance (ANCOVA) model was performed to assess whether the association between CTRP-1 levels and PCOS remained significant after adjustment for these potential confounding variables. In this model, CTRP-1 level was treated as the dependent variable, PCOS status was included as a fixed factor, and age and BMI were included as covariates.

HOMA-IR data were unavailable for one participant, and biochemical hyperandrogenism data were unavailable for two participants. Analyses involving these variables were therefore performed using available-case analysis without imputation.

All statistical tests were two-tailed, and p-values <0.05 were considered statistically significant.

## 3 Results

This study included a total of 96 women, comprising 48 patients diagnosed with PCOS and 48 healthy controls, who were recruited from the Infertility Outpatient Clinic of Ankara City Hospital between October 2021 and March 2022. The ages of the participants ranged from 17 to 38 years, with a mean age of 25.6 years. The mean age was significantly lower in the PCOS group compared with the control group (23.6 vs. 27.7 years, respectively; p < 0.001).

Body weight and BMI were significantly higher in the PCOS group than in the control group (p < 0.001 for both comparisons). Additionally, the distribution of obesity differed significantly between the PCOS and control groups (p = 0.016). The anthropometric and demographic characteristics of the study population are summarized in Table 1.

**Table 1:** Baseline demographic and anthropometric characteristics of the study groups.

Variable	PCOS (n = 48)	Control (n = 48)	Total (n = 96)	p value
Age (years)	22.0 (20.0–26.0)	27.0 (25.0–31.0)	25.0 (21.5–28.0)	<0.001^a^
Weight (kg)	75.0 (63.0–84.5)	59.0 (55.0–69.0)	65.0 (58.0–80.0)	<0.001ᵃ
BMI (kg/m²)	27.7 (24.4–30.8)	22.5 (20.6–27.4)	25.3 (21.3–29.2)	<0.001ᵃ
**WHO Obesity Classification (BMI)**				0.016^b^
Underweight (<18.5)	0 (0.0%)	1 (2.1%)	1 (1.0%)	
Normal weight (18.5–24.9)	16 (33.3%)	30 (62.5%)	46 (47.9%)	
Overweight (25–29.9)	18 (37.5%)	11 (22.9%)	29 (30.2%)	
Obese (30–39.9)	13 (27.1%)	6 (12.5%)	19 (19.8%)	
Morbidly obese (≥40)	1 (2.1%)	0 (0.0%)	1 (1.0%)	

Values are presented as **median (interquartile range)** or **n (%)**.

^a^Mann–Whitney U test

^b^Fisher–Freeman–Halton test

**Abbreviations:** BMI: Body Mass Index; PCOS: Polycystic Ovary Syndrome; WHO: World Health Organization.

**Fig 1:**
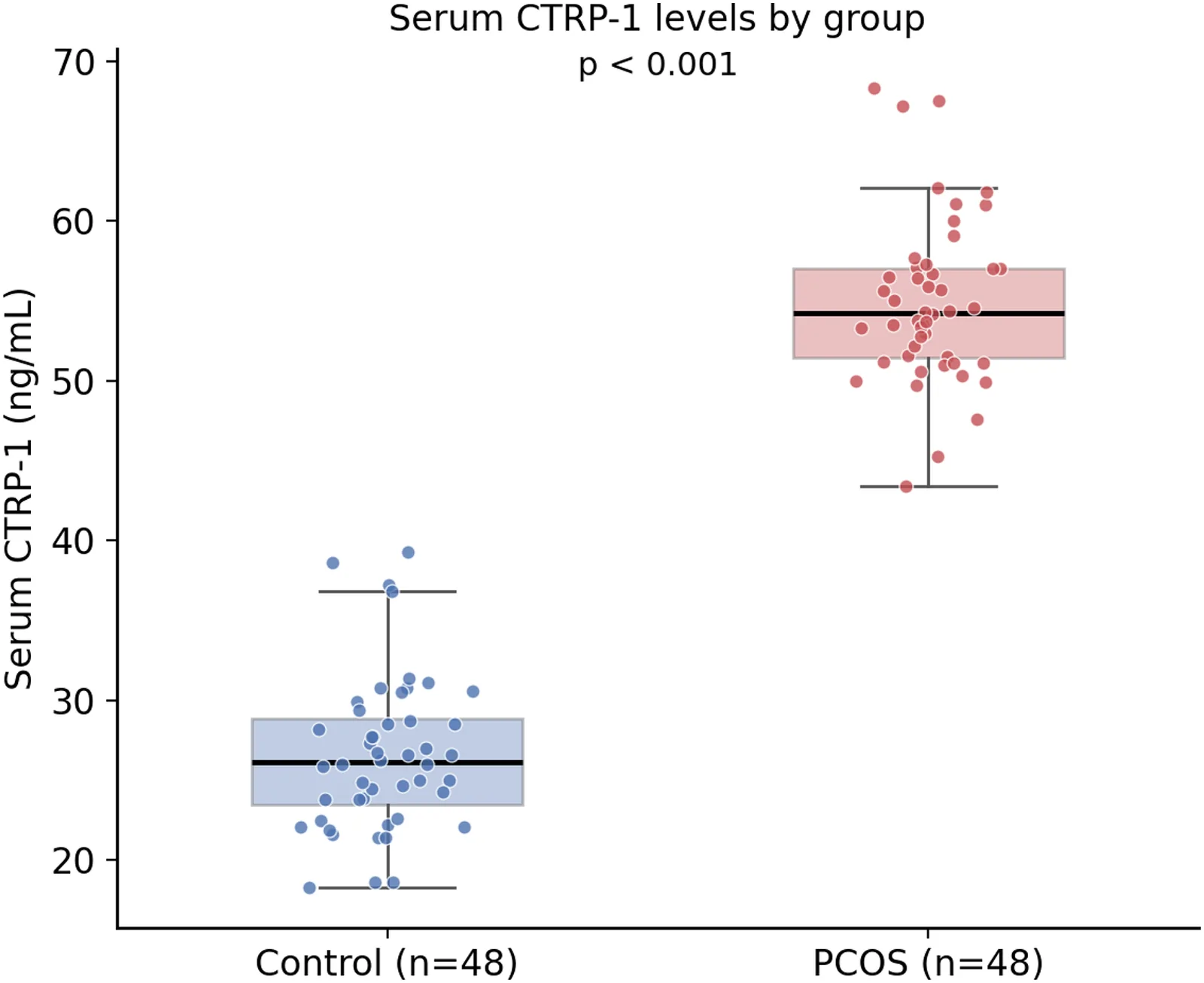
Distribution of serum CTRP-1 levels in the PCOS and control groups. Boxes show the median and interquartile range; individual data points are overlaid to display the distribution of values within each group and the separation between groups (Mann–Whitney U test, p < 0.001).

Serum CTRP-1 levels were significantly higher in the PCOS group compared with the control group (p < 0.001). The mean CTRP-1 level was 54.9 ng/mL in the PCOS group and 26.6 ng/mL in the control group (Table 2, Fig 1). CTRP-1 concentrations used for statistical analyses are provided in S2 Dataset.

**Table 2:** Comparison of CTRP-1 levels between the PCOS and control groups.

Variable	PCOS (n = 48)	Control (n = 48)	p value
CTRP-1 (ng/mL)	54.9 ± 5.2; 54.3 (51.4–57.1)	26.6 ± 4.8; 26.2 (23.2–29.0)	<0.001^a^

Values are presented as **mean±SD and median (interquartile range)**.

^a^Student’s t-test.

**Abbreviations:** PCOS: Polycystic Ovary Syndrome.

**Fig 2:**
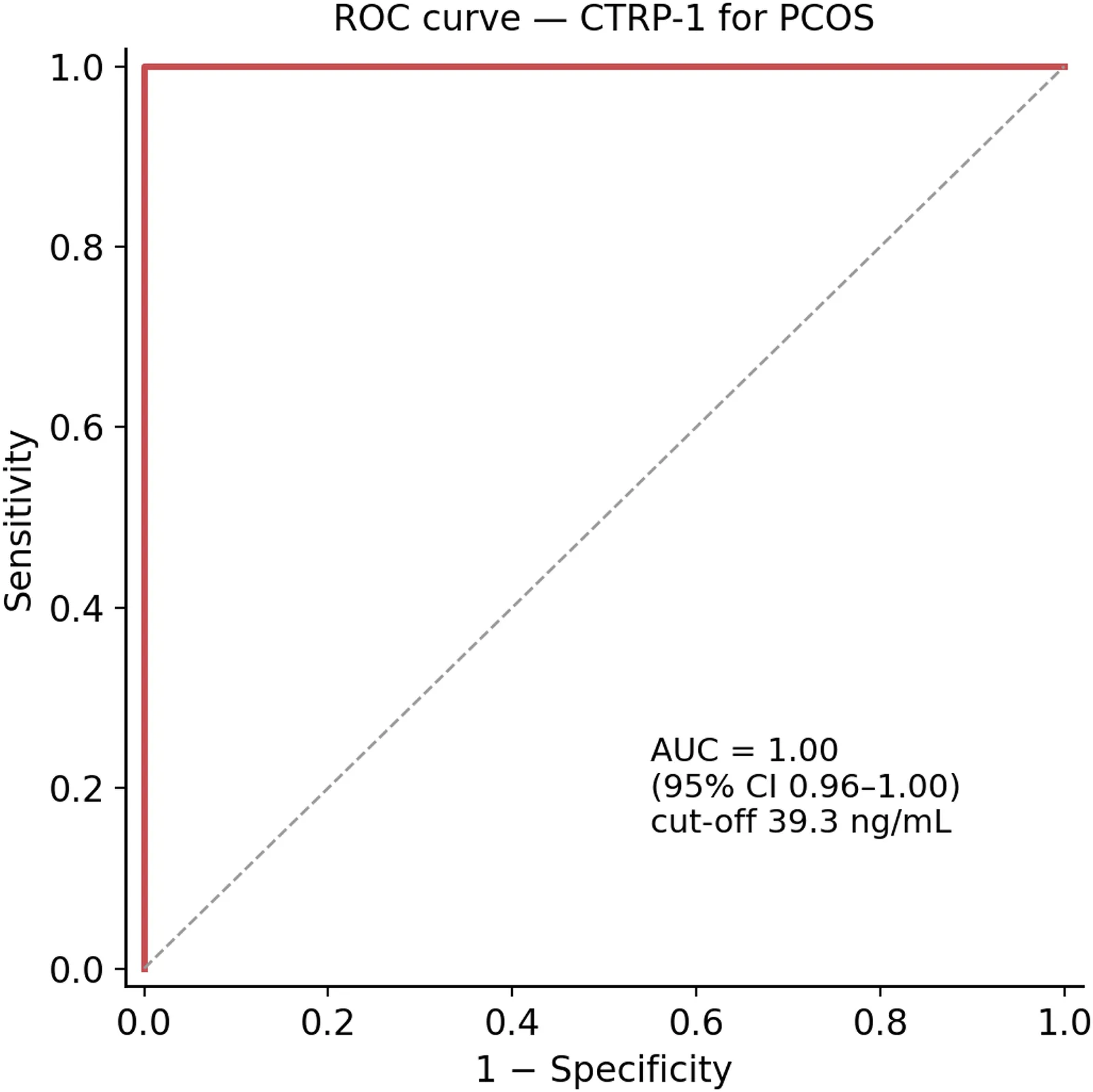
Receiver operating characteristic (ROC) curve analysis of CTRP-1 for the diagnosis of PCOS. AUC = 1.00 (95% CI 0.96–1.00); optimal cut-off 39.3 ng/mL.

Receiver operating characteristic (ROC) curve analysis was performed to evaluate the discriminatory ability of CTRP-1 for identifying PCOS within the present cohort. The optimal cut-off value for CTRP-1 was 39.3 ng/mL. At this threshold, the calculated sensitivity and specificity were both 100%, with an area under the curve (AUC) of 1.00 (Fig 2, Table 3).

**Table 3:** Receiver operating characteristic (ROC) analysis of CTRP-1 for discrimination of PCOS within the present cohort.

Variable	AUC (95% CI)	Cut-off	Sensitivity	Specificity
CTRP-1	1.00 (0.96–1.00)	39.3 ng/mL	100% (92.6–100)	100% (92.6–100)*

*Findings should be interpreted cautiously because the ROC analysis was derived and evaluated within the same dataset and may overestimate real-world diagnostic performance.

**Fig 3:**
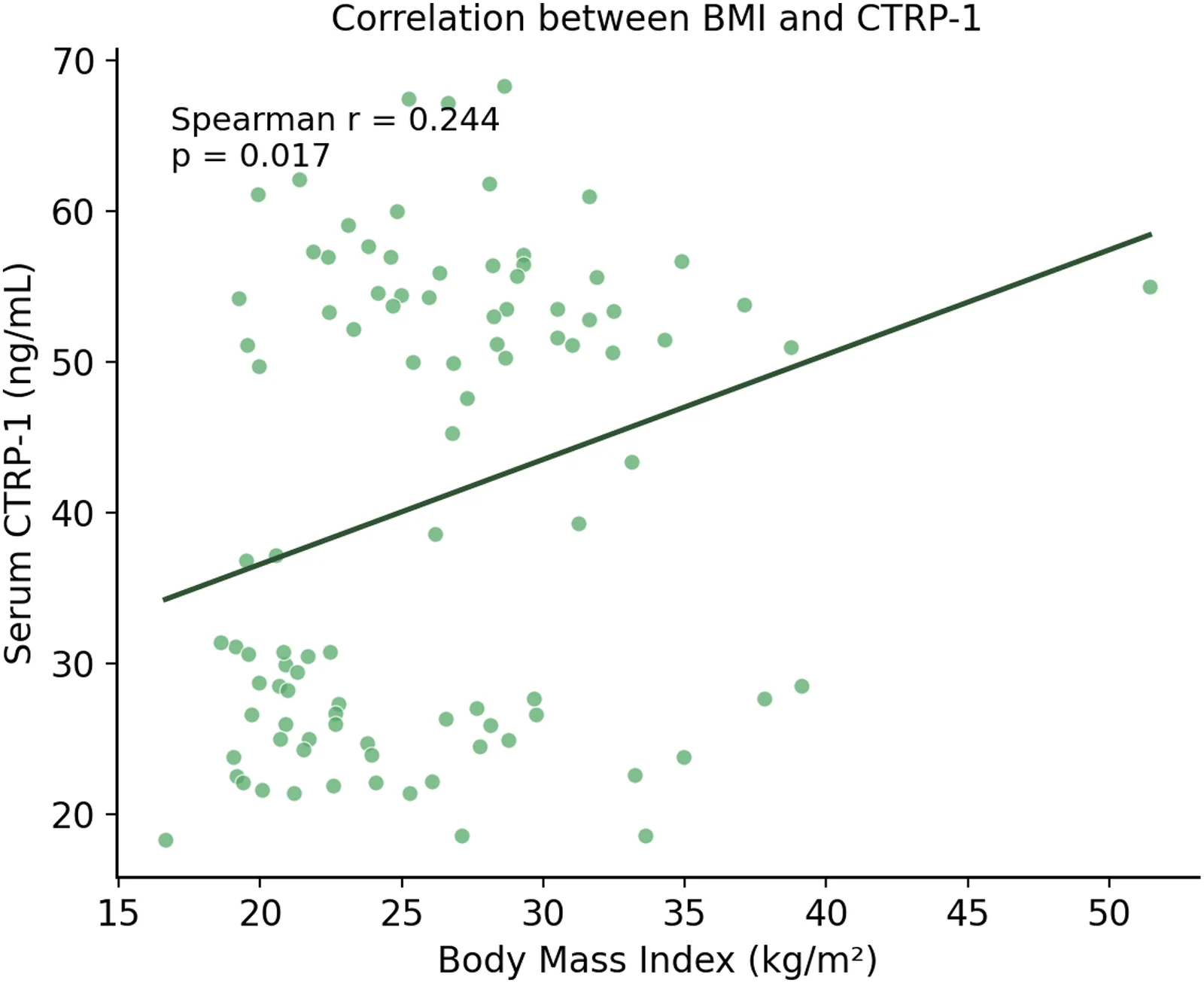
Correlation between body mass index (BMI) and serum CTRP-1 levels (Spearman r = 0.244, p = 0.017).

However, these findings should be interpreted cautiously given the relatively small sample size and the case–control design of the study, which may increase the risk of overestimation of diagnostic performance. The observed separation between groups reflects the characteristics of this specific cohort and should therefore be considered preliminary and hypothesis-generating pending external validation in larger and more heterogeneous populations. The dataset used for ROC curve construction, including individual CTRP-1 concentrations and group classifications, is provided in S2 Dataset to allow independent evaluation of the analysis. Internal validation confirmed the robustness of this separation within the present cohort: bootstrap resampling (2000 iterations) yielded a 95% confidence interval of 1.00–1.00 for the AUC, the optimism-corrected AUC was 1.00, and repeated five-fold cross-validation produced a mean AUC of 1.00. At the optimal cut-off, both sensitivity and specificity were 100% (95% CI 92.6–100%). A logistic model based on age and BMI alone yielded a cross-validated AUC of 0.80, which increased to 1.00 after the addition of CTRP-1, indicating that CTRP-1 provided discriminatory information beyond age and BMI. The individual distribution of CTRP-1 concentrations in the two groups, illustrating the complete separation between PCOS patients and controls, is displayed together with the group summaries in Fig 1. Correlation analyses were conducted to evaluate the relationship between CTRP-1 levels and demographic, anthropometric, and metabolic parameters. A weak but significant negative correlation was observed between age and CTRP-1 levels (r = −0.379, p < 0.001), indicating that CTRP-1 levels decreased with increasing age. Conversely, BMI showed a weak but significant positive correlation with CTRP-1 levels (r = 0.244, p = 0.017). Similarly, body weight demonstrated a weak positive correlation with CTRP-1 levels (r = 0.244, p = 0.017) (Fig 3). The relationships between CTRP-1 levels and demographic and anthropometric variables are presented in Table 4.

**Table 4:** Correlation between CTRP-1 levels and demographic and anthropometric parameters.

Variable	r	p value
Age	−0.379	<0.001**
BMI (kg/m²)	0.244	0.017*
Body Weight (kg)	0.244	0.017*

r = Spearman’s correlation coefficient.

*p < 0.05.

**p < 0.01.

**Abbreviations:** BMI: Body Mass Index.

**Fig 4:**
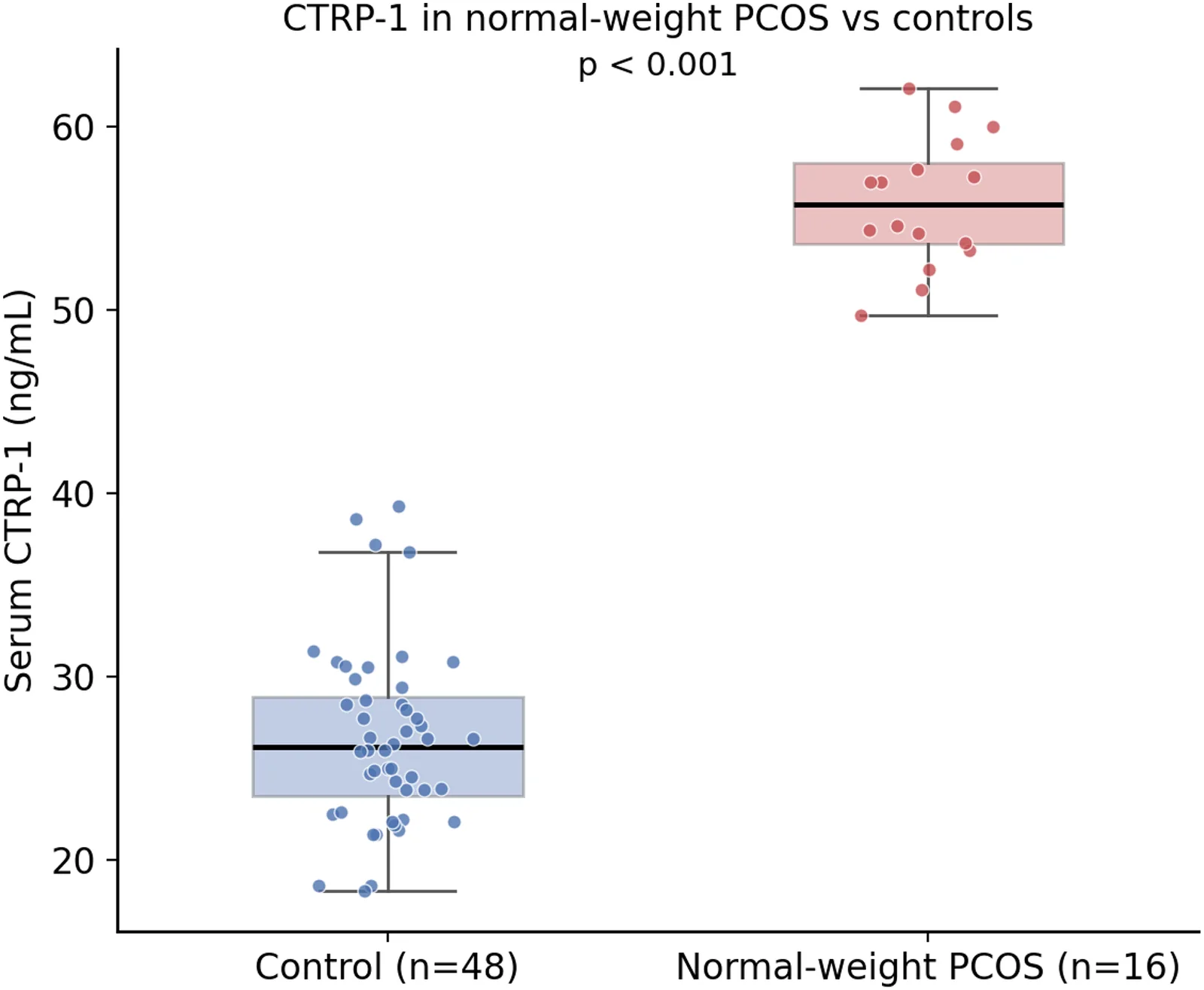
Distribution of serum CTRP-1 levels in normal-weight PCOS patients (n = 16) and the control group (n = 48). Boxes show the median and interquartile range with individual data points overlaid (Mann–Whitney U test, p < 0.001).

When PCOS patients were evaluated according to the World Health Organization obesity classification, no underweight individuals were identified in the PCOS group. Among participants with normal BMI, CTRP-1 levels were significantly higher in normal-weight PCOS patients compared with controls (p < 0.001) (Table 5, Fig 4).

**Table 5:** Evaluation of CTRP-1 levels in normal-weight PCOS patients and the control group.

Groups	CTRP-1 (ng/mL) Mean±SD	Median (Q1–Q3)	p value
Normal-weight PCOS patients (n = 16)	55.9 ± 3.6	55.8 (53.5–58.4)	<0.001**
Control (n = 48)	26.6 ± 4.8	26.2 (23.2–29.0)	
Total (n = 64)	33.9 ± 13.6	28.0 (24.4–44.5)	

ᶜ Student’s t-test.

**p < 0.01.

**Abbreviations:** PCOS: Polycystic Ovary Syndrome.

To further account for baseline differences in age and BMI, a 1:1 nearest-neighbour matched analysis was performed within the normal-weight stratum, matching each PCOS patient to a control participant on age and BMI (16 matched pairs). CTRP-1 levels remained significantly higher in PCOS patients than in their matched controls (55.9 ± 3.6 vs. 26.3 ± 3.5 ng/mL; Wilcoxon signed-rank p = 0.0004), with no overlap between the two groups.

To determine whether the association between CTRP-1 levels and PCOS was independent of potential confounding variables, an analysis of covariance (ANCOVA) was performed with age and body mass index included as covariates.The results demonstrated that the presence of PCOS remained independently associated with higher CTRP-1 levels (β = 28.97, p < 0.001). In contrast, neither age (p = 0.648) nor BMI (p = 0.228) showed a significant independent effect on CTRP-1 levels. These findings indicate that the elevated CTRP-1 concentrations observed in the PCOS group may not be explained solely by differences in age or adiposity.

Further analysis demonstrated significant associations between CTRP-1 levels and several metabolic parameters. CTRP-1 levels showed a weak positive correlation with fasting insulin levels (r = 0.209, p = 0.043). In addition, a moderate positive correlation was found between anti-Mullerian hormone (AMH) levels and CTRP-1 (r = 0.433, p < 0.001). Conversely, HDL cholesterol levels were negatively correlated with CTRP-1 levels (r = −0.296, p = 0.003). A weak positive correlation was also observed between triglyceride levels and CTRP-1 (r = 0.233, p = 0.022). These associations are presented in Table 6. After correction for multiple comparisons using the Benjamini–Hochberg procedure, the correlations of CTRP-1 with age, AMH, and HDL cholesterol remained statistically significant (FDR-adjusted p < 0.05), whereas the weaker correlations with BMI, body weight, fasting insulin, and triglycerides did not retain significance after adjustment and are therefore regarded as exploratory.

**Table 6:** Correlation between CTRP-1 levels and biochemical variables.

Variable	r	p value
Total Testosterone	0.197	0.059
Glucose	0.120	0.245
Fasting Insulin	0.209	0.043*
HOMA-IR	0.152	0.144
HbA1c	0.094	0.395
DHEA-S	0.065	0.538
AMH	0.433	<0.001**
Total Cholesterol	−0.089	0.386
LDL	−0.087	0.398
HDL	−0.296	0.003**
VLDL	0.198	0.054
Triglycerides	0.233	0.022*

r = Spearman’s correlation coefficient.

*p < 0.05.

**p < 0.01.

HOMA-IR values were available for 95 participants. Insulin resistance was detected in 27 participants, whereas 68 participants did not exhibit insulin resistance. The mean CTRP-1 level was significantly higher in participants with insulin resistance compared with those without insulin resistance (50.8 ng/mL vs. 36.6 ng/mL, p < 0.001).

Further exploratory analyses were performed to evaluate CTRP-1 levels according to individual clinical and biochemical features of PCOS, including hirsutism, menstrual irregularity, biochemical hyperandrogenism, and polycystic ovarian morphology. Participants presenting with these features generally demonstrated higher CTRP-1 concentrations compared with those without these findings. However, because these subgroup analyses were based on diagnostic components of PCOS itself, the findings should be interpreted cautiously. Detailed exploratory subgroup analyses are presented in S4 Dataset.

## 4 Discussion

Polycystic ovary syndrome (PCOS) is a complex endocrine and metabolic disorder characterized by insulin resistance, hyperinsulinemia, and ovarian hyperandrogenism resulting from dysregulation of the hypothalamic–pituitary–ovarian axis. Affecting approximately 6–10% of women of reproductive age, PCOS is clinically manifested by hirsutism, oligo-anovulation, and polycystic ovarian morphology [1,4]. In addition to reproductive dysfunction, PCOS is frequently accompanied by metabolic abnormalities such as obesity, dyslipidemia, and impaired glucose tolerance, which may increase the long-term risk of subfertility, type 2 diabetes mellitus, metabolic syndrome, and cardiovascular disease [3–5].

The present study demonstrated that circulating CTRP-1 levels were significantly higher in women with PCOS compared with healthy controls. To the best of our knowledge, this is the first study evaluating circulating CTRP-1 concentrations in women with PCOS and examining their association with metabolic and reproductive parameters. These findings suggest that CTRP-1 may be involved in the metabolic and endocrine alterations associated with the syndrome.

Although no previous studies have directly evaluated CTRP-1 in PCOS, several investigations have examined other members of the CTRP family. Previous studies reported decreased circulating levels of CTRP3, CTRP5, CTRP12, and CTRP13 in women with PCOS [23]. These reductions were associated with insulin resistance, obesity, and androgen excess. In contrast, other CTRP family members such as CTRP6 and CTRP15 have been reported to be elevated in PCOS, suggesting a potential compensatory response to metabolic inflammation and insulin resistance [24,25]. Taken together, these findings indicate that CTRP family proteins may play heterogeneous but important roles in the metabolic disturbances associated with PCOS. Within this context, the elevated CTRP-1 levels observed in our study may represent another component of this adipokine network involved in PCOS pathophysiology.

Experimental evidence suggests that CTRP-1 participates in several metabolic signaling pathways relevant to PCOS. CTRP-1 has been associated with AMP-activated protein kinase (AMPK) activation, regulation of glucose uptake through Akt-mediated GLUT-4 translocation, and modulation of fatty acid oxidation and inflammatory pathways [13,17–21]. Because insulin resistance and chronic low-grade inflammation are considered central mechanisms in PCOS, elevated CTRP-1 levels may reflect a compensatory metabolic response associated with these processes.

Receiver operating characteristic (ROC) curve analysis demonstrated complete separation of CTRP-1 concentrations between the PCOS and control groups within this cohort. However, this finding should be interpreted cautiously. The relatively small sample size, single-center case–control design, and the homogeneity of the study population may have contributed to an overestimation of diagnostic performance. In addition, because the optimal cut-off value and diagnostic metrics were derived and evaluated within the same dataset, the reported AUC may not accurately reflect real-world clinical performance. The observed complete separation between groups reflects the characteristics of this specific cohort and should therefore be considered preliminary and hypothesis-generating pending external validation in larger, prospective, and more heterogeneous populations.

An important limitation of the present study is the baseline difference in age and BMI between the PCOS and control groups. Women in the PCOS group were younger and had higher BMI values compared with controls. Since CTRP-1 levels demonstrated correlations with both age and BMI, these differences may have partially influenced the observed associations. Although ANCOVA analysis demonstrated that PCOS status remained independently associated with CTRP-1 levels after adjustment for age and BMI, residual confounding cannot be completely excluded. Furthermore, although a significant negative correlation between age and CTRP-1 levels was observed in univariate analysis, age did not remain independently associated with CTRP-1 levels in the adjusted ANCOVA model. This discrepancy may reflect the influence of interrelated covariates together with the relatively limited sample size of the present study.

One of the most important findings of the present study was that CTRP-1 levels remained significantly elevated even among normal-weight women with PCOS. Although obesity and adiposity are closely linked to adipokine regulation, this subgroup analysis suggests that the observed elevation in CTRP-1 may not be solely attributable to increased body mass and may instead reflect underlying endocrine or metabolic disturbances intrinsic to PCOS itself. This observation may support the hypothesis that CTRP-1 is associated not only with obesity-related metabolic dysfunction, but also with mechanisms more directly related to PCOS pathophysiology. Nevertheless, because this subgroup analysis was exploratory and based on a relatively limited sample size, larger age- and BMI-matched studies are required to confirm these findings.

Correlation analyses revealed a weak but significant negative association between CTRP-1 levels and age. However, this finding should be interpreted cautiously because the control group was older than the PCOS group. CTRP-1 levels also demonstrated positive correlations with BMI, body weight, fasting insulin, triglyceride levels, and AMH concentrations, whereas HDL cholesterol levels were negatively correlated with CTRP-1. After correction for multiple comparisons, only the associations with age, AMH, and HDL cholesterol remained statistically significant, whereas the correlations with BMI, body weight, fasting insulin, and triglycerides did not and are therefore interpreted as hypothesis-generating. Of note, BMI and body weight yielded identical Spearman correlation coefficients owing to their very high collinearity (Spearman’s rho = 0.93), which produces near-identical rank orderings; the corresponding Pearson coefficients differ slightly (r = 0.27 and r = 0.29, respectively). These findings are generally consistent with previous studies reporting associations between CTRP family members and insulin resistance, adiposity, and cardiometabolic risk factors [23,26,27]. Similar elevations in circulating CTRP-1 concentrations have also been reported in metabolic disorders including type 2 diabetes mellitus, coronary artery disease, and metabolic syndrome [22].

Direct comparison of absolute CTRP-1 concentrations across studies is limited by methodological heterogeneity, particularly the use of different ELISA platforms. In previous reports, circulating CTRP-1 concentrations in control participants have generally been higher than those observed in the present study, ranging from approximately 150 ng/mL in healthy women [28] to a median of around 233 ng/mL in a mixed-sex cohort [23] and approximately 308 ng/mL in healthy men [19]; reported concentrations have also been consistently lower in women than in men [23,28]. In our cohort, control CTRP-1 levels averaged 26.6 ng/mL, measured with a different commercial assay (CLOUD-CLONE; limit of detection 8.3 ng/mL). These lower absolute values most likely reflect differences in assay calibration and dynamic range rather than a true biological discrepancy, since the direction of change—elevation in metabolic disease and, in the present study, in PCOS—is concordant across studies. This inter-assay variability also implies that the optimal cut-off derived here is assay-specific and should not be transferred directly to other platforms without recalibration, further reinforcing the need for external validation.

We also observed a moderate positive correlation between CTRP-1 and anti-Müllerian hormone (AMH) levels. Since AMH reflects ovarian follicle number and is commonly elevated in women with PCOS, this association may indicate a possible relationship between CTRP-1 and ovarian follicular dysfunction. Similarly, higher CTRP-1 levels observed in women with hirsutism, menstrual irregularity, and polycystic ovarian morphology further support a relationship between CTRP-1 and the reproductive manifestations of PCOS. However, because these subgroup analyses were exploratory and based on diagnostic features of PCOS itself, these findings should be interpreted cautiously.

## 5 Strengths and limitations

The present study has several strengths. First, to the best of our knowledge, this is the first study investigating circulating CTRP-1 levels in women with PCOS. Second, all participants were evaluated using the same standardized clinical, biochemical, and laboratory protocol, which improved consistency of data collection and analysis. Third, in addition to evaluating CTRP-1 concentrations, we investigated their associations with a broad range of anthropometric, metabolic, and hormonal parameters, allowing a more comprehensive assessment of the potential relationship between CTRP-1 and PCOS-related metabolic dysfunction. Furthermore, the inclusion of a normal-weight PCOS subgroup provided additional insight into the relationship between CTRP-1 and PCOS independent of obesity, representing an important proof-of-concept finding within the study.

Several limitations should also be acknowledged. First, the study population was relatively small and recruited from a single tertiary referral center, which may limit the generalizability of the findings. Second, the cross-sectional case–control design precludes causal interpretation and may increase the risk of overestimating diagnostic performance measures. In addition, significant differences in age and BMI between the PCOS and control groups may have introduced residual confounding despite statistical adjustment using ANCOVA. Although CTRP-1 levels remained independently associated with PCOS after adjustment, residual confounding cannot be completely excluded.

Another important limitation is that the ROC analysis was both derived and evaluated within the same dataset. Therefore, the observed complete separation between groups and the resulting AUC value should be interpreted cautiously and considered preliminary and hypothesis-generating rather than definitive evidence of clinical diagnostic utility. External validation in larger, prospective, multicenter cohorts with more heterogeneous populations is necessary before any clinical application can be considered.

Although CTRP-1 concentrations were measured using a commercially available ELISA kit with an established detection range and sensitivity, analytical variability inherent to biomarker assays should be considered when interpreting measurements obtained near the lower range of detection.

In addition, multiple exploratory correlation and subgroup analyses were performed, which may increase the possibility of type I error. Because subgroup analyses according to individual PCOS features were exploratory and based on diagnostic components of PCOS itself, these findings should also be interpreted cautiously.

## 6 Conclusion

In conclusion, circulating CTRP-1 levels were significantly elevated in women with PCOS and were associated with several metabolic and reproductive characteristics of the syndrome. Elevated CTRP-1 concentrations were observed even among normal-weight women with PCOS, suggesting that this association may not be explained solely by obesity. Although the present findings support a possible relationship between CTRP-1 and the metabolic dysregulation underlying PCOS, the diagnostic performance observed in this cohort should be interpreted cautiously. Larger prospective multicenter studies are needed to validate these findings and to clarify the biological mechanisms linking CTRP-1 with PCOS pathophysiology.

## Supporting information

S1 Dataset Complete dataset including demographic, clinical, and biochemical variables. (XLSX)

S2 Dataset Processed CTRP-1 concentrations used for statistical analyses. (XLSX)

S3 Dataset Raw ELISA output file including optical density values, calibration data, and calculated concentrations. (XLSX)

S4 Dataset Exploratory subgroup analyses of CTRP-1 levels according to individual PCOS features. (DOCX)
