# Correction: Circulating CTRP-1 levels are elevated in women with polycystic ovary syndrome: A case–control study

**DOI:** 10.1371/journal.pone.0362136

**Published:** 2026-11-03

**Authors:** Cavide Ali, Alper Şayir, Feride Öçba, Deniz Öztürk Atan, Melike Sevde Harmancı, Mustafa Cem Sürecek, Hacer Cavidan Gülerman

There is an error in affiliation 3 for author Mustafa Cem Sürecek. The correct affiliation 3 is: Department of Anesthesiology and Reanimation, Çanakkale State Hospital, Çanakkale, Turkey.
