# Supplementary material for: Circulating CTRP-1 levels are elevated in women with polycystic ovary syndrome: A case–control study
Source: PLoS One. 2026 Aug 25;21(8):e0356461. doi: 10.1371/journal.pone.0356461 (PMC13505912; doi:10.1371/journal.pone.0356461)
Supplement: Supplementary file 1 [file pone.0356461.s004.docx]

Supplementary Material 4

Exploratory subgroup analyses of CTRP-1 levels according to individual PCOS features

| **Variable** | **Group** | **CTRP-1 (ng/mL) Mean±SD** | **Median (Q1–Q3)** | **p value** |
| --- | --- | --- | --- | --- |
| **Insulin Resistance** | Present (n=27) | 50.8±9.9 | 53.7 (51.0–55.9) | <0.001** |
|  | Absent (n=68) | 36.6±15.0 | 29.7 (24.8–51.9) |  |
| **Hirsutism** | Present (n=18) | 53.6±4.6 | 54.0 (51.5–57.0) | <0.001** |
|  | Absent (n=78) | 37.8±15.1 | 30.6 (25.0–53.3) |  |
| **Biochemical Hyperandrogenism** | Present (n=11) | 47.8±11.5 | 52.2 (45.3–55.0) | 0.050* |
|  | Absent (n=83) | 39.5±15.3 | 31.4 (25.9–53.8) |  |
| **Menstrual Irregularity** | Present (n=45) | 53.0±9.7 | 54.2 (51.1–57.0) | <0.001** |
|  | Absent (n=51) | 30.0±9.7 | 27.0 (23.9–30.8) |  |
| **Polycystic Ovary Morphology** | Present (n=44) | 55.2±5.0 | 54.3 (51.4–57.2) | <0.001** |
|  | Absent (n=52) | 28.6±8.5 | 26.6 (23.8–30.6) |  |

ᶜ Student’s *t*-test

*p < 0.05
**p < 0.01
